# Measuring health-related quality of life in multiple sclerosis: comparing the acceptability, validity and responsiveness of the EQ-5D-3L and MSIS-8D

**DOI:** 10.1007/s11136-026-04229-5

**Published:** 2026-04-01

**Authors:** Elizabeth Goodwin, Bernhard Michalowsky, Rod Middleton, Annie Hawton

**Affiliations:** 1 University of Exeter Medical School, University of Exeter, Exeter, UK; 2 Patient-Reported Outcomes and Health Economics Research, German Center for Neurodegenerative Diseases (DZNE), Greifswald, Germany; 3 Department of Health Research Methods, Evidence and Impact, McMaster University, Hamilton, Canada; 4 Population Data Science, Swansea University, Swansea, UK

**Keywords:** Preference-based measures, Health-related quality of life, Psychometrics, Economic evaluation, Multiple sclerosis, EQ-5D

## Abstract

**Purpose:**

Concerns have been raised about the sensitivity and responsiveness of the EQ-5D, one of the most commonly used preference-based health-related quality of life measures, in the context of multiple sclerosis (MS). In response to these concerns, a condition-specific preference-based measure, the Multiple Sclerosis Impact Scale Eight Dimensions (MSIS-8D), was developed. This research aimed to assess the psychometric and distributional properties of the MSIS-8D compared to the EQ-5D-3L, in people with MS.

**Methods:**

Analyses were undertaken using data from the UK MS Register. Both measures were compared in terms of acceptability (missing data), distributional properties (health state frequencies, health state density curves and indices), construct validity in relation to disability, mobility, fatigue, anxiety and depression (discriminative and convergent validity, using ANOVA, independent t-tests and Spearman correlations), and responsiveness to symptom onset and relapse (mean change scores, standardised response means, standardised effect sizes, paired t-tests).

**Results:**

The MSIS-8D exhibited superior distributional properties, while the EQ-5D-3L showed greater acceptability. Both measures demonstrated excellent construct validity. Neither measure appeared responsive to symptom onset, and only the MSIS-8D met all criteria for responsiveness when people moved from a non-relapse to a relapse state.

**Conclusion:**

Although the MSIS-8D appears to offer superior distributional properties and responsiveness compared to the EQ-5D-3L, the responsiveness of both measures in this analysis was limited. This adds weight to existing concerns about the ability of utility measures used in healthcare decision-making to fully capture treatment effects in MS.

## Abstract (plain language summary)

In the UK, healthcare treatments must be shown to be cost-effective in order to be provided on the NHS. When assessing cost-effectiveness, researchers usually measure the effects of treatments on people’s health using a questionnaire called the EQ-5D. However, concerns have been raised about the ability of the EQ-5D to capture the effects of treatments for multiple sclerosis. This could affect decisions about which new treatments are made available to people with multiple sclerosis. Another questionnaire, called the MSIS-8D, has been developed specifically to measure the effects of treatments on the health of people with multiple sclerosis. Evidence is needed to assess whether the MSIS-8D is better able to capture these treatment effects in people with multiple sclerosis, compared to the EQ-5D. In this study, we used data collected by the UK Multiple Sclerosis Register to compare the EQ-5D and the MSIS-8D. We compared their relevance to people with multiple sclerosis, their ability to distinguish between groups of people with more or less severe symptoms, and their ability to capture changes in people’s health over time. The results indicate that the MSIS-8D is more relevant to people with multiple sclerosis and better able to capture changes in people’s health over time, compared to the EQ-5D. However, both the EQ-5D and the MSIS-8D showed limited ability to capture changes over time. This suggests that more research is required to understand how the effects of treatments for multiple sclerosis can be better measured, and fully represented in assessments of cost-effectiveness.

## Introduction

MS is the most common, non-traumatic cause of disability among younger adults worldwide [1]. It is a complex and progressive condition, affecting the central nervous system [2]. This causes a wide range of physical, psychological and cognitive symptoms, which vary considerably across individuals [3]. The most common subtype is relapsing–remitting MS (RRMS), in which the disease course is characterised by periods of relapse and remission. The majority of people with RRMS go on to develop secondary progressive MS (SPMS). Around 10–15% of people with MS are diagnosed with primary progressive MS (PPMS), which is progressive from the outset. Levels of disability increase as the disease progresses [2]. Research has shown that MS patients show considerable decrements compared to the general population on all domains of health-related quality of life (HRQoL) [4].

The National Institute for Health and Care Excellence (NICE) recommends the use of the EQ-5D, a generic preference-based measure (PBM) of HRQoL, for use in economic evaluations of new healthcare interventions in England and Wales [5]. Following a number of unfavourable decisions by NICE regarding the cost-effectiveness of disease-modifying treatments (DMTs) for multiple sclerosis (MS), concerns were raised about the appropriateness of using the EQ-5D-3L to measure health outcomes in MS [6], particularly in relation to its content and construct validity [7–10]. Also, evidence for the responsiveness of the EQ-5D to changes in the HRQoL of people with MS is lacking [10], which is of particular concern in the context of economic evaluation, given that a measurement tool with poor responsiveness may fail to demonstrate effects of treatment when they occur.

When undertaking economic evaluations of treatments for conditions in which the EQ-5D lacks validity and responsiveness, it can be acceptable to use a condition-specific preference-based measure (CSPBM) [11]. The descriptive system for a CSPBM includes dimensions of HRQoL that are of particular relevance to people with that condition, and may exclude less relevant domains that are typically included in generic GPBMs. This enhanced content validity aims to more fully capture important differences and changes in HRQoL, however this can come at a cost [12]. Consistent use of the EQ-5D allows direct comparability of economic evaluation results across treatments and conditions. While use of the same valuation protocol will achieve a degree of comparability between PBMs with different descriptive systems, CSPBMs may be less likely to capture side-effects and impacts of comorbidities, and their associated utility values may be prone to focusing effects during valuation due to the lack of a broader context around condition-specific health state descriptions [13]. For these reasons, it is generally recommended that CSPBMs are used in addition to, rather than instead of, a generic PBM [12].

In response to the concerns regarding the appropriateness of the EQ-5D for MS, a CSPBM, the Multiple Sclerosis Impact Scale-Eight Dimensions (MSIS-8D), was developed, enabling utility values to be estimated from responses to an existing patient-reported measure of HRQoL in MS, the Multiple Sclerosis Impact Scale (MSIS-29-v2) [14]. The MSIS-8D tariff was estimated using preferences from a sample of the UK general population via the time trade-off method [15]. The MSIS-8D has been used alongside the EQ-5D in trial-based economic evaluations [16–19]. However, there is still limited evidence for whether the MSIS-8D demonstrates the intended improvements in acceptability, validity and responsiveness compared to the EQ-5D.

Therefore, this study aimed to assess the acceptability, distributional properties, construct (discriminative and convergent) validity and responsiveness of the MSIS-8D, compared to the EQ-5D-3L, in a large, representative cohort of people with MS living in the UK, in order to inform decisions regarding the appropriate choice of outcome measures for economic evaluations of treatments for MS. The content of this paper is informed by the revised COSMIN reporting guideline for studies on measurement properties of patient-reported outcome measures [20].

## Methods

### UK MS register and population

This analysis used data routinely collected by the UK MS Register (UKMSR), a large, ongoing, prospective, longitudinal, observational, cohort study that was launched in 2011 [21]. Recruitment to the UKMSR is by word of mouth, information provided to patients of the UKMSR’s clinical partner sites (currently 56 NHS hospitals across the UK), or presentations and display stands at relevant events. At Spring 2023, the number of current, consented UKMSR members was c10,600, with around 54% of these providing full MSIS-29v2 data at this timepoint. The membership of the UKMSR has been shown to be broadly representative of people living with MS in the UK [22, 23].

On registration, new members are requested to provide information on socio-demographic and health-related characteristics and to complete a number of patient-reported outcome measures (PROMs). Subsequently, all members receive email reminders to complete the PROMs on a regular basis. From May 2011 to October 2017, members were invited to provide data on a 3-monthly basis, for the MSIS-29v2, EQ-5D-3L, Multiple Sclerosis Walking Scale (MSWS-12), and Hospital Anxiety and Depression Scales (HADS-A, HADS-D). From October 2017 onwards, this changed to 6-monthly data collection, and the self-reported Expanded Disability Status Score (web-EDSS) and Fatigue Severity Scale (FSS) were added to the suite of PROMs. The PROMs are presented as a ’to do’ list with members able to choose their own order of completion. Members are prompted to update their socio-demographic and health-related characteristics data annually. All data entry is online, via a secure internet portal [21].

Participants must be aged 18 or over, and provide consent via a Terms of Service agreement [23]. Ethical approval for the UKMSR has been provided by the South West Central Bristol Research Ethics Council initially as 16/SW/0164 now 21/SS/0085.

Cross-sectional analyses were based on data from all UKMSR members providing data on both the EQ-5D-3L and MSIS-29 at the Spring 2023 data collection time-point. Responsiveness analysis used data from UKMSR members who reported experiencing either one of two events (described below) that would be expected to have an effect on their HRQoL, up to the Spring 2023 data collection window. The UKMSR restricted the data extracts for both analyses to people who had provided full responses to the EQ-5D-3L at Spring 2023 and on at least four more occasions over the 11 year period.

### Data and instruments

The ‘target measures’ for the analysis were:

- EQ-5D-3L: a generic, preference-based measure of HRQoL with five dimensions (mobility, self-care, usual activities, pain/discomfort, anxiety/depression) and three response levels (1 = no, 2 = some, 3 = extreme problems), generating 243 health states. Respondents are asked to describe their health ‘today’. Responses to the EQ-5D-3L were converted to utility values using the UK value set, which has a maximum value range from −0.594 to 1.000 [24].
- MSIS-8D: an MS-specific preference-based measure of HRQoL with eight dimensions taken from items of the MSIS-29v2 (physical tasks, social activities, mobility, daily activities, fatigue, emotion, cognition, depression) and four response levels (1 = not at all, 2 = a little, 3 = moderately, 4 = extremely), generating 65,536 health states. Respondents are asked to describe the impact of their MS on each dimension over the past two weeks. The MSIS-8D descriptive system is presented in the Appendix. MSIS-29v2 responses were converted to MSIS-8D utility values by the application of a published algorithm based on the preferences of a representative sample of the general UK population, which has a maximum value range from 0.079 to 0.882 [15].

The variables representing events expected to affect HRQoL were date of most recent relapse, and date of onset of two or more new symptoms. The criteria for selection of these events were (i) hypothesised to have an effect on HRQoL and (ii) recorded on the UKMSR with sufficient detail to enable responsiveness analysis. Further details of these variables and hypotheses are provided in Table 1.

**Table 1:** Description of variables representing events expected to affect HRQoL, their hypothesised effects on HRQoL, and how they were used in the responsiveness analyses

Relapse	
Hypothesis	We would expect HRQoL during a relapse to be lower than that experienced during a period of remission. Recovery following relapse is often incomplete, resulting in a sustained negative impact on quality of life for up to 12 months [29], hence the most relevant comparison will be between pre-relapse and during-relapse utility values
Rationale	MS relapses are characterised by an exacerbation of existing MS symptoms, or the appearance of new symptoms, for a period of time, followed by complete or partial remission. People with MS report that relapses impact upon their daily activities, emotional wellbeing, social functioning and work performance [30]
Variable description	Participants who have answered “yes” to “Have you had ANY relapses in the last 6 months?” are asked to report the calendar month during which the most recent relapse occurred. The relevant year can be inferred from the auto-generated date-stamp for this variable. These data were used to create a variable that identifies the month and year in which the relapse occurred
Time-points	EQ-5D-3L and MSIS-8D utility values for an assessment point [*t*–1] *prior* to the reported relapse were compared with EQ-5D-3L and MSIS-8D utility values for the *same assessment point* [*t*] in which the relapse was reported, provided that no current relapse was reported at the date of the earlier utility value [*t*–1]
Inclusion criteria	Participants were included in the analyses if: • The calendar month and year of their most recent relapse were the same as the calendar month and year of reporting a utility value and • They reported a utility value at a preceding time-point [*t*–1] within 12 months (400 days) prior to the date at which the utility value corresponding to the relapse was reported and • They did not report a current relapse at [*t*–1] A participant could be included more than once in the analysis if they met the above inclusion criteria at more than one point during the study period
Notes:	The actual number of days used to represent one year was increased to 400 days to allow for variation in the point during each data collection window at which the individual MSR member completed the questionnaires

Onset of new symptoms	
Hypothesis	We would expect the onset of new symptoms to result in an immediate deterioration in HRQoL
Rationale	A variety of individual physical, psychological and cognitive symptoms of MS have been found to have a significant, negative impact on the quality of life of people with MS [31]
Variable description	Participants are asked to report which symptoms they experience from a list of 25 symptoms, and to report the date of onset of each symptom
Time-points	EQ-5D-3L and MSIS-8D utility values for an assessment point [*t*–1] *prior* to the symptom onset date were compared with EQ-5D-3L and MSIS-8D utility values for an assessment point [*t*] *up to 30 days after* the symptom onset date
Inclusion criteria	Participants were included in the analyses if: • They reported the onset of a new symptom and the reported date of symptom onset was within the study period and • They reported a utility value within one year (400 days) prior to the reported start date of the symptom and • They reported a utility value up to 1 month (30 days) after the reported start date of the symptom and • They reported the onset of at least two new symptoms between the dates of the two utility values For people reporting the onset of a particular symptom more than once in the study period, only the first reported date of symptom onset was included. People could be included more than once in the analysis if they reported the onset of two or more symptoms (according to the inclusion criteria above) more than once during the assessment period
Other details	Symptoms: optic neuritis, double vision, impairment of motor control, sensory loss, pins and needles, muscle pain, bladder problems, bowel problems, sexual dysfunction, altered sensation, weakness, spasticity, difficulty swallowing, difficulty speaking, trigeminal neuralgia, tremors, dysarthia, nystagmus, fatigue, depression, pain, cognitive difficulties, brief repetitive symptoms, gait, ataxia

Data for analysis also included socio-demographics (age, gender, ethnicity), MS subtype (RRMS, SPMS, PPMS, benign MS) and the following standardized and validated PROMs: the web-EDSS, which measures MS-specific disability [25]; the FSS [26]; the MSWS-12 [27]; and the HADS-A and HADS-D [28].

### Statistical analyses

#### Cross-sectional analyses

Acceptability was assessed by comparing rates of missing data for the target measures, using a separate data extract that included all people who responded to at least one of the UKMSR’s regularly administered survey instruments during the Spring 2023 data collection window. The UKMSR data collection portal does not allow partial responses to instruments. Therefore information on acceptability at an individual dimension level was not available.

The distributional properties of the EQ-5D-3L and MSIS-8D descriptive systems were compared by examining the frequency of health states across the sample including floor and ceiling effects, health state density curves (HSDCs) and health state density indices (HSDIs). The HSDC and HSDI are analogous to the Lorenz curve and Gini coefficient that are frequently used to describe income distributions [32]. HSDCs provide a graphical representation of how evenly responses to a measure are distributed across the full range of possible health state profiles. Total equality of distribution, i.e. a hypothetical sample in which the same proportion of participants report each health state, is represented by a 45% line. The closer the HSDC is to the 45% line, the more evenly participants are distributed across health states. The HSDI ranges from 0 to 1, where 1 represents total equality of distribution and 0 represents total inequality (i.e. all participants reported the same health state) [33]. In addition, bar charts were used to illustrate the distribution of responses across individual dimension levels.

The discriminative validity of the target measures was evaluated according to their ability to differentiate between subgroups based on levels of disability (web-EDSS) and symptom severity (MSWS-12, FSS, HADS), assuming an inverse relationship between disability or symptom severity and HRQoL. The published cut-off scores for the webEDSS [25], FSS [34], HADS-D and HADS-A [28], and the mid-point score for the MSWS-12 [35], were used to create binary groups for levels of disability, fatigue, depression, anxiety and walking impairment. The target measures were also compared according to their ability to differentiate between MS subtypes, assuming that HRQoL diminishes from benign MS to RRMS, PPMS and SPMS [36]. Differences in mean utility values between subgroups were assessed using one-way ANOVAs or independent t-tests.

Convergent validity was assessed by examining Spearman’s correlation coefficients between the target measures and each of the measures of disability (web-EDSS) and symptom severity (MSWS-12, FSS, HADS). A correlation coefficient between 0.3 and 0.5 represents a moderate relationship; strong relationships are considered to be ≥ 0.5 [37].

#### Longitudinal analysis (responsiveness)

The responsiveness of the target measures was assessed by examining changes in utility values from before to during a relapse, and from before to after onset of new symptoms. Paired t-tests were used to indicate the presence or absence of the hypothesised effect on HRQoL, and to provide an initial signal of responsiveness. Standardised effect sizes (SES), calculated as the mean change divided by the standard deviation of the earlier mean score, were used to determine whether the change on either instrument was statistically non-negligible, i.e. at least a small effect size (SES ≥ 0.2) [38]. Standardised response means (SRM), calculated as the mean change divided by the standard deviation of the mean change, were used to compare responsiveness between the EQ-5D-3L and MSIS-8D, by estimating 95% confidence intervals for the SRMs [39, 40]. Scores on individual dimensions of the target measures before and after reported changes in each of the selected variables were compared using the Wilcoxon Signed Rank Test for paired samples.

All analyses were undertaken in Stata 18.

## Results

### Sample characteristics

Data from 3676 UKMSR members were available for the cross-sectional analyses. The characteristics of this sample are summarised in Table 2.

**Table 2:** Descriptive statistics for cross-sectional analysis

Cross-sectional analysis		
	Obs	Percent
Included in analysis		
Observations	3676	
Participants	3676	
Gender		
Female	2727	74.18
Male	946	25.73
Ethnicity		
White British	3365	91.79
Other/not stated	311	8.21
Contemporary MS type^a^		
RRMS	1862	50.67
SPMS	1110	30.2
PPMS	491	13.36
Benign^b^	76	2.07
Not known	136	3.7

Other variables	Obs	Mean	S.D	Min	Max
Age^a^	3676	58.32	10.8	22	89
webEDSS score	3619	4.94	2.06	0	9
MSWS-12 total score	2854	44.99	32.72	0	100
FSS total score	3667	4.85	1.52	1	7
HADS-D total score	3625	6.64	4.27	0	21
HADS-A total score	3616	6.89	4.48	0	21
EQ-5D-3L value	3676	0.567	0.321	− 0.594	1
MSIS-8D value	3676	0.626	0.185	0.079	0.882

Obs: observations; S.D.: standard deviation; RRMS: relapsing–remitting MS; SPMS: secondary progressive MS; PPMS: primary progressive MS

^a^MS Type and Age are contemporary with each data collection window (i.e. Age increases over time and MS Type changes if amended by the UKMSR member)

^b^Benign MS is characterised by minimal physical disability maintained over a duration of 10 or more years following diagnosis

### Acceptability

Of the 6141 UKMSR members who were included in the acceptability (missing data) analysis, 3.14% did not complete the EQ-5D-3L (n = 5948) and 8.57% did not complete the MSIS-8D (n = 5615). Differences were statistically significant (*p* < 0.001).

### Distributional properties

The EQ-5D-3L exhibited a significantly greater ceiling effect than the MSIS-8D (409 observations, 11.13% versus 142 observations, 3.86%; *p* < 0.001). Both instruments had negligible floor effects (< 1%). The most frequently observed EQ-5D-3L health state was 22222 (493 observations, 13.41%). The most frequently observed MSIS-8D health state was 11111111 (142 observations, 3.86%), representing the instrument's ceiling. Nearly half the observations in the sample (49.32%) were covered by five EQ-5D-3L health states: 22222, 11111, 22221, 21222, 21221. Conversely, the five most frequently observed MSIS-8D health states accounted for only 8.19% of the sample: 11111111, 21111111, 11112221, 11112111, 11112121.

**Fig. 1:**
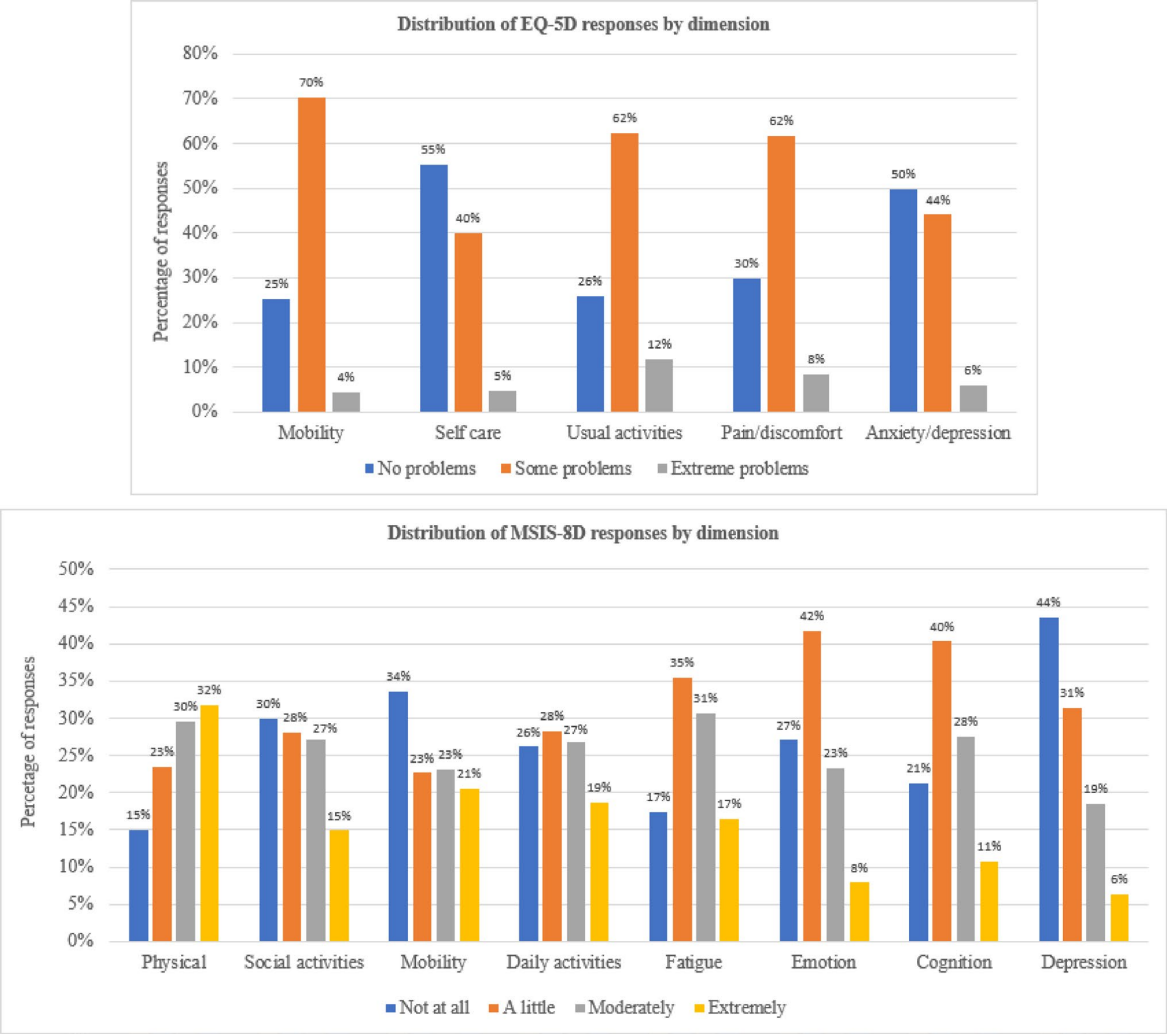
Distribution of EQ-5D-3L and MSIS-8D responses by dimension

Figure 1 illustrates the distribution of EQ-5D-3L and MSIS-8D responses by dimension. Very low proportions of responses were observed at Level 3 of all EQ-5D-3L dimensions. The EQ-5D-3L *Mobility*, *Usual Activities* and *Pain/Discomfort* dimensions show a high concentration of responses at Level 2, and *Self-care* at Level 1. Responses were more evenly distributed across each MSIS-8D dimension than they were for the EQ-5D-3L, although the proportions at the ‘extremely’ level for the *Emotion*, *Cognition* and *Depression* dimensions were relatively low, with higher proportions reporting ‘not at all’ for *Depression* and ‘a little’ for *Emotion* and *Cognition*.

**Fig. 2:**
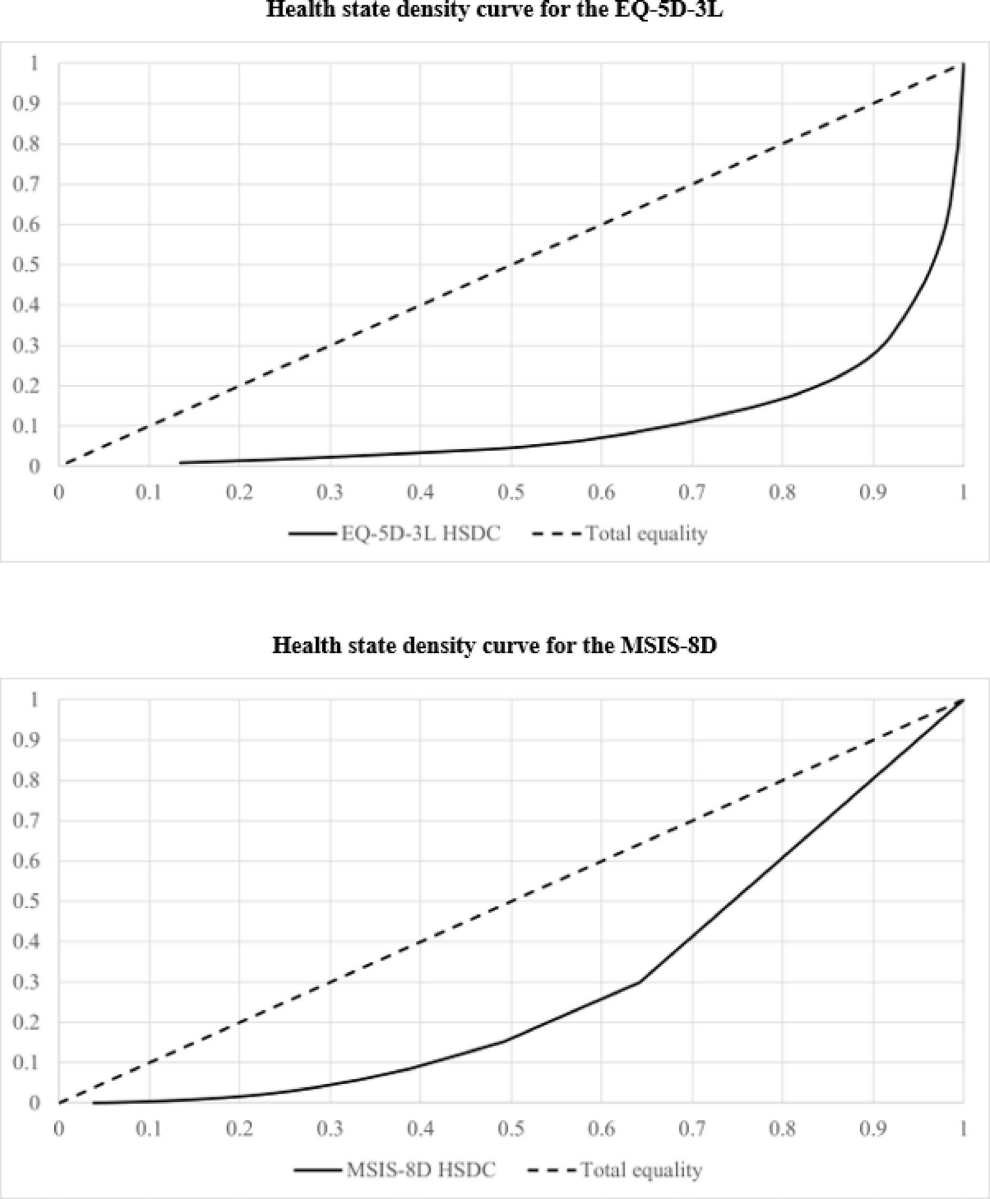
Health state density curves for the EQ-5D-3L and MSIS-8D

The HSDCs for the EQ-5D-3L and MSIS-8D (Fig. 2) show that the MSIS-8D HSDC is closer to the dotted line, which represents total equality of distribution, than the EQ-5D-3L HSDC. The HSDI was closer to one for the MSIS-8D than for the EQ-5D (MSIS-8D = 0.577; EQ-5D-3L = 0.215).

### Construct validity

Table 3 presents the results for discriminative and convergent validity. Both target measures significantly discriminated between groups based on levels of disability, fatigue, depression, anxiety, walking impairment and type of MS (*p* < 0.0001). The EQ-5D-3L and MSIS-8D utility values showed strong correlations (rho > 0.5) with the disability, fatigue, walking and depression measures, and moderate correlations with the anxiety measure (rho > 0.4), with very high statistical significance (*p* < 0.0001). While the EQ-5D-3L showed a slightly higher correlation with the disability measure and the walking scale, the MSIS-8D better correlated with the anxiety, depression and fatigue scales.

**Table 3:** Discriminative and convergent validity of the EQ-5D-3L and MSIS-8D

EQ-5D-3L	Discriminative validity						Convergent validity	
	Obs	Mean	S.D	t-stat	df	*p* value*	Rho	*p* value*
Fatigue								
FSS < 4	923	0.773	0.233	24.307	3665	< 0.0001	− 0.553	< 0.0001
FSS > = 4	2744	0.498	0.316					
Walking								
MSWS < 50	1548	0.764	0.214	31.474	2852	< 0.0001	− 0.677	< 0.0001
MSWS > = 50	1306	0.469	0.284					
Depression								
HADS-D < 11	2959	0.629	0.281	26.921	3623	< 0.0001	− 0.586	< 0.0001
HADS-D > = 11	666	0.290	0.345					
Anxiety								
HADS-A < 11	2811	0.621	0.293	19.947	3614	< 0.0001	− 0.414	< 0.0001
HADS-A > = 11	805	0.378	0.345					
Disability								
webEDSS < 5	1719	0.745	0.221	37.490	3617	< 0.0001	− 0.704	< 0.0001
webEDSS > = 5	1900	0.406	0.311					

MS type				F-stat	*p* value*
RRMS	1862	0.674	0.277	204.100	< 0.0001
SPMS	1110	0.414	0.324		
PPMS	491	0.483	0.308		
Benign	76	0.773	0.281		

MSIS-8D	Discriminative validity						Convergent validity	
	Obs	Mean	S.D	t-stat	df	*p* value*	Rho	*p* value*
Fatigue								
FSS < 4	923	0.771	0.114	30.996	3665	< 0.0001	− 0.651	< 0.0001
FSS > = 4	2744	0.577	0.178					
Walking								
MSWS < 50	1548	0.741	0.129	33.316	2852	< 0.0001	− 0.669	< 0.0001
MSWS > = 50	1306	0.553	0.172					
Depression								
HADS-D < 11	2959	0.668	0.158	32.797	3623	< 0.0001	− 0.668	< 0.0001
HADS-D > = 11	666	0.440	0.182					
Anxiety								
HADS-A < 11	2811	0.666	0.161	26.224	3614	< 0.0001	− 0.493	< 0.0001
HADS-A > = 11	805	0.488	0.196					
Disability								
webEDSS < 5	1719	0.724	0.141	34.985	3617	< 0.0001	− 0.634	< 0.0001
webEDSS > = 5	1900	0.538	0.175					

MS type				F-stat	*p* value*
RRMS	1862	0.684	0.168	184.250	< 0.0001
SPMS	1110	0.542	0.177		
PPMS	491	0.581	0.173		
Benign	76	0.752	0.148		

S.D.: standard deviation; df: degrees of freedom; RRMS: relapsing-remitting MS; SPMS: secondary progressive MS; PPMS: primary progressive MS

*Bonferroni correction gives adjusted significance level of *p* < 0.000962

### Responsiveness

The characteristics of the UKMSR members included in each of the four analyses (EQ-5D-3L and MSIS-8D responsiveness to symptom onset and relapse) are summarised in Table 4.

**Table 4:** Descriptive statistics for longitudinal analysis samples

EQ-5D-3L analysis						
	Relapses			Symptom onset		
	Obs	Percent		Obs	Percent	
Included in analysis						
Observations	156			97		
Participants	142			93		
Gender						
Female	125	80.13		71	0.73	
Male/not reported	31	19.87		26	0.27	
Ethnicity						
White British	142	91.03		88	0.91	
Other/not stated	14	8.97		9	0.09	
Contemporary MS type^a^						
RRMS	125	80.13		56	0.58	
Other/not known	24	15.38		41	0.42	

Other variables	Obs	Mean	S.D	Obs	Mean	S.D
Age (years)^a^	156	52.37	12.47	97	54.74	10.47
Web-EDSS^b^	85	4.79	1.83	61	5.39	1.64
MSWS-12	114	43.32	30.8	53	57.32	28.27
FSS^b^	148	5.13	1.54	96	5.52	1.17
HADS depression	151	7.88	5	97	8.62	4.36
HADS anxiety	151	8.48	5.23	97	7.80	4.83
EQ-5D-3L value	156	0.507	0.34	97	0.444	0.318
MSIS-8D value	152	0.573	0.21	97	0.532	0.192

MSIS-8D analysis						
	Relapses			Symptom onset		
	Obs	Percent		Obs	Percent	
Included in analysis						
Observations	152			95		
Participants	138			92		
Gender						
Female	125	82.24		72	0.76	
Male/not reported	27	17.76		23	0.24	
Ethnicity						
White British	139	91.45		85	0.89	
Other/not stated	13	8.55		10	0.11	
Contemporary MS type^a^						
RRMS	120	78.95		53	0.56	
Other/not known	26	17.11		42	0.44	

Other variables	Obs	Mean	S.D	Obs	Mean	S.D
Age (years)^a^	152	52.33	12.3	95	54.78	10.17
Web-EDSSb	86	4.83	1.86	60	5.56	1.56
MSWS-12	108	43.63	30.69	49	58.94	27.50
FSS^b^	144	5.22	1.55	93	5.58	1.15
HADS depression	148	7.91	4.86	94	8.69	4.19
HADS anxiety	148	8.39	5.17	94	7.84	4.76
EQ-5D-3L value	147	0.493	0.35	93	0.441	0.306
MSIS-8D value	152	0.567	0.21	95	0.536	0.183

Obs: observations; S.D.: standard deviation; RRMS: relapsing-remitting MS

^a^MS Type and Age are contemporary with each data collection window

^b^The webEDSS and FSS were introduced by the UKMSR in 2017, resulting in a lower number of observations for these compared to the other measures

Table 5 shows the results of the responsiveness analyses for the EQ-5D-3L and MSIS-8D for each of the two events. When comparing utility values during relapse with those at an earlier time point at which no relapse was present, only the MSIS-8D produced a significant or non-negligible change (i.e., *p* < 0.001; SES > 0.2). The SRM for the MSIS-8D was also significantly higher than the EQ-5D-3L at a 95% confidence level. Neither instrument met the threshold for a significant or non-negligible change in response to the onset of two or more symptoms, although the SRM for the MSIS-8D was significantly higher than for the EQ-5D-3L. No statistically significant changes were observed in the individual dimensions of the EQ-5D-3L or MSIS-8D in response to either relapse or symptom onset.

**Table 5:** Responsiveness results

								SRM				Direction of change				*p* values	
	Obs	Mean	SD	t-stat	df	*p* val	SES	SRM	LCL	UCL		+ ve	− ve	None	z-stat	Actual	Exact
Current relapse																	
EQ-5D-3L											Dimension						
Change score	156	− 0.047	0.208	2.821	155	0.0054	− 0.139	− 0.226	− 0.259	− 0.193	MB	<10	<10	136	−2.236	0.253	
Pre-relapse	156	0.554	0.338								SC	<10	<10	139	− 0.243	0.8084	
Relapse	156	0.507	0.342								UA	13	21	122	− 1.362	0.1733	0.2145
Participants	142										PD	10	23	123	− 2.263	0.0236	0.0351
											AD	15	25	116	− 1.581	0.1138	0.1539
MSIS-8D											Dimension						
Change score	152	− 0.047	0.142	4.067	151	0.0001*	− 0.228	− 0.330	− 0.352	− 0.307	Phys	23	45	84	− 2.723	0.0065	0.0067
Pre-relapse	152	0.614	0.205								Soc	36	45	71	− 0.911	0.3622	0.3707
Relapse	152	0.567	0.214								Mob	25	47	80	− 2.636	0.0084	0.0080
Participants	138										DA	26	48	78	− 2.780	0.0054	0.0049
											Fat	19	37	96	− 2.381	0.0173	0.0192
											Emo	23	47	82	− 2.829	0.0047	0.0049
											Cog	24	39	89	− 1.994	0.0462	0.0469
											Dep	26	43	83	− 2.049	0.0405	0.0415
Onset of two or more new symptoms																	
EQ-5D-3L											Dimension						
Change score	97	− 0.022	0.232	0.9307	96	0.3544	− 0.069	− 0.094	− 0.141	− 0.048	MB	<10	<10	80	−1.698	0.0896	
Pre-onset	97	0.466	0.318								SC	<10	<10	81	−1.500	0.1336	
Post-onset	97	0.444	0.318								UA	14	13	70	0.160	0.8726	1.0000
Participants	93										PD	10	19	68	− 1.671	0.0947	0.1360
											AD	12	16	69	− 0.783	0.4338	0.4744
MSIS-8D											Dimension						
Change score	95	− 0.034	0.161	2.0653	94	0.0416	− 0.190	− 0.212	− 0.244	− 0.179	Phys	11	27	57	− 2.734	0.0063	0.0059
Pre-onset	95	0.570	0.180								Soc	24	33	38	− 1.246	0.2128	0.2291
Post-onset	95	0.536	0.183								Mob	25	29	41	− 0.495	0.6204	0.6270
Participants	92										DA	20	35	40	− 2.097	0.0360	0.0377
											Fat	18	32	45	− 2.089	0.0367	0.0384
											Emo	23	27	45	− 0.467	0.6404	0.6601
											Cog	16	30	49	− 2.122	0.0338	0.0368
											Dep	20	25	50	− 0.628	0.5303	0.5242

Key: Statistical terms (from left to right): Obs: Observations, SD: standard deviation, df: degrees of freedom, *p*-val: *p* value, SES: standardised effect size, SRM: standardised response mean, LCL: lower confidence limit, UCL: upper confidence limit, + ve: positive (improvement), − ve: negative (deterioration), *significant after Bonferroni correction (*p* < 0.000962), Cell counts < 10 suppressed. EQ-5D-5L dimensions: MB: Mobility, SC: Self-care, UA: Usual activities, PD: Pain/discomfort, AD: Anxiety/depression

## Discussion

This study provides, for the first time, an assessment of the acceptability, validity and responsiveness of the MSIS-8D, compared to the EQ-5D-3L. This evidence, based on a large, representative dataset, provides essential information to address the current lack of knowledge regarding the validity and responsiveness of these measures in people with MS. The use of a range of analysis methods, exploring the descriptive systems of the measures as well as utility values, allows interpretations and potential explanations to be drawn from across the results.

### Cross-sectional analyses

The higher rate of missing data for the MSIS-8D (8.57%) compared to the EQ-5D-3L (3.14%) suggests that the EQ-5D-3L is more acceptable to respondents with MS. This may be due to the longer length of the MSIS-29 questionnaire, from which MSIS-8D values are derived, with 29 items compared to the EQ-5D-3L’s five.

The EQ-5D-3L exhibited a high concentration of relatively few health states. The most commonly reported EQ-5D-3L health state was 22222, which might suggest the need for greater discrimination between the extremes of ‘no problems’ and ‘severe problems’—an issue that has been addressed with the introduction of the EQ-5D-5L [41]. This is supported by the high ceiling effect that was observed for the EQ-5D-3L, indicating a lack of sensitivity at higher levels of HRQoL. The results from the HSDCs and HSDIs indicate that participants are more evenly distributed across MSIS-8D health states than across EQ-5D-3L health states, suggesting that the MSIS-8D is better able to differentiate between participants [33]. This is likely due to the higher number of unique health states described by the MSIS-8D classification system (65,536) compared to the EQ-5D-3L (243), as well as its condition-specific content.

Responses across the levels of each dimension were more evenly distributed for the MSIS-8D than they were for the EQ-5D-3L. Proportions of responses at the ‘extremely’ level of the MSIS-8D *Emotion*, *Cognition* and *Depression* dimensions were relatively low, however, with more than 40% of responses at Level 2 (‘a little*’*) for *Emotion* and *Cognition* and at Level 1 (‘not at all*’*) for *Depression*. This may indicate that the definitions of these dimension levels are limited in their ability to distinguish between degrees of functioning or symptom severity that are important to people with MS. However, the more even distribution of responses for the MSIS-8D overall may, in part, explain its greater responsiveness compared to the EQ-5D-3L.

Both measures exhibited excellent discriminative validity, with statistically significant differences concerning MS type, and levels of disability, walking impairment, fatigue, depression and anxiety. This supports the ability of the EQ-5D-3L and MSIS-8D to distinguish between groups known to differ in terms of HRQoL. In terms of convergent validity, both the EQ-5D-3L and MSIS-8D correlated strongly with measures of disability, fatigue, walking impairment and depression, and moderately with anxiety. The EQ-5D-3L correlated most strongly with measures related to walking; in addition to the MSWS-12, the webEDSS is also primarily focused on ambulatory function [25]. This may be because the mobility item of the EQ-5D-3L focuses specifically on ‘walking’, whereas the MSIS-8D mobility item does not. The MSIS-8D correlated more strongly with anxiety, depression and fatigue. These findings may be informative when selecting outcome measures to evaluate interventions targeting specific MS symptoms.

### Responsiveness analysis

Changes in EQ-5D-3L and MSIS-8D utility values were assessed in relation to relapse and the onset of two or more symptoms. The only statistically significant and/or non-negligible change was for the MSIS-8D in response to relapse, however the SES was small (−0.228). Given the results of other studies, which have found that relapses [29, 30] and individual MS symptoms [31] have a considerable impact on HRQoL, these findings may suggest a lack of responsiveness for both instruments. This may be unsurprising for the EQ-5D-3L, given the concerns previously raised about its sensitivity to the effects of MS treatment. These concerns primarily relate to content and construct validity, particularly the omission of HRQoL domains important to people with MS, such as fatigue and cognitive problems [10], as well as ceiling effects and limited convergent validity [7]. This is only partially supported by the current analysis, which identified high ceiling effects for the EQ-5D-3L but excellent convergent validity for both target measures. Furthermore, despite its low ceiling effect and inclusion of fatigue and cognition dimensions, the MSIS-8D also exhibited limitations in responsiveness, although the SRMs for the MSIS-8D were higher than those for the EQ-5D-3L in both analyses.

The MSIS-8D descriptive system was designed to be more sensitive to changes in MS-specific HRQoL, with dimensions selected to represent aspects of HRQoL of most relevance to people with MS [11]. This is reflected in the greater content validity exhibited by the MSIS-8D compared to the EQ-5D-3L, as well as its greater responsiveness when assessed using standardised measures (SES and SRM). When using a preference-based measure in economic evaluation, however, it is the magnitude of change in mean values that is relevant. In the current analysis, the mean change in relation to relapse was identical for both instruments (EQ-5D-3L = −0.047, MSIS-8D = −0.047), whereas there was a marked difference in relation to symptom onset (EQ-5D-3L = −0.022, MSIS-8D = −0.034). The former may be due to the narrower potential value range of the MSIS-8D (0.079–0.882) compared to the EQ-5D-3L (− 0.594–1.000) restricting the potential size of changes in values [36]. Hence any advantages in responsiveness gained from the MSIS-8D’s condition-specific descriptive system may be off-set by its narrower range of utility values.

### Limitations of the study

During the time period covered by these analyses, the UKMSR administered the EQ-5D-*3L*, introducing the EQ-5D-*5L* in Autumn 2023. The EQ-5D-5L retains the same five dimensions as the EQ-5D-3L, with the number of response options per dimension increased from three to five. This increases the potential sensitivity of the descriptive system to more subtle changes in each dimension, which could improve responsiveness and reduce ceiling effects [41]. Findings from previous studies that directly compared the EQ-5D-3L and EQ-5D-5L support the improved sensitivity of the EQ-5D-5L [42–45]. Clearly, the results of this study will not reflect any such improvement. The extent to which the shift from three to five levels for the same dimensions will increase sensitivity to change in MS, where the relevance of the EQ-5D dimensions themselves has been the main point of contention [10], remains to be seen.

The use of pre-existing datasets in research is increasing due to a number of advantages, including increased efficiency and reduced participant burden. The disadvantage of this approach, however, is that the available data were not designed to address the given research question [46]. In the current study, while the UKMSR data proved highly suitable for the cross-sectional analyses, the assessment of responsiveness was more challenging. This was partly due to the need to identify a specific event, occurring at a known point in time, that could be expected to have a detectable, positive or negative, effect on HRQoL. Such clear-cut events were difficult to identify in the UKMSR data, limiting the analyses that could be performed. The recorded timing of events relied on participant self-report, potentially introducing measurement error and temporal misclassification, and the length of time between the reported date of events and a preceding or subsequent utility value was determined by the UKMSR’s fixed three or 6 monthly data collection points, preventing exploration of shorter-term effects. Further to this, previous analysis of UKMSR data shows that responses to PROMs peak at 5–10 years post-diagnosis, reducing monotonically over time thereafter, potentially resulting in under-representation of people with more advanced MS [47]. The dataset provided by the UKMSR included only participants who provided EQ-5D-3L responses for at least five timepoints, including complete EQ-5D-3L responses at Spring 2023, thereby reducing the overall number of participants included in the analyses. This may have resulted in under-representation of particular groups, potentially introducing survivorship and selection bias. The two events (relapse, symptom onset) were each considered in isolation; the responsiveness analyses do not control for other events occurring during the relevant time frame that might have influenced HRQoL. For these reasons, the results of the responsiveness analyses reported here should be interpreted with some caution.

## Conclusions

The MSIS-8D demonstrated superior content validity and distributional properties to the EQ-5D-3L, while the EQ-5D-3L showed greater acceptability in this sample of people with MS living in the UK. Both measures exhibited good construct validity. However, neither measure was responsive to the onset of new symptoms, and only the MSIS-8D met all criteria for responsiveness when people moved from a non-relapse to a relapse state. These findings support earlier concerns regarding the sensitivity of the EQ-5D to important changes in the lives of people with MS, and suggest that similar issues may affect the MSIS-8D, albeit to a lesser extent. The responsiveness results, however, should be interpreted with some caution due to a number of limitations that affected the analysis, several of which arose due to the use of existing data. Future studies could usefully explore how best use can be made of valuable data resources such as the UKMSR in longitudinal research, potentially alongside the prospective collection of bespoke data. Further research is also needed to assess the distributional and psychometric properties of the EQ-5D-5L in this population.

## Figures and Tables

**Fig. 3:**
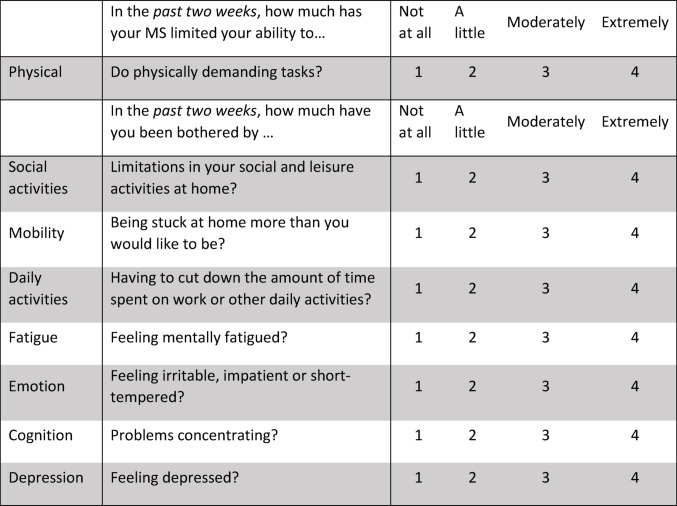
Descriptive system for the MSIS-8D

## Data Availability

The datasets used in this study are stored in the UK MS Register Secure e-Research platform. These data can be accessed by suitably qualified researchers following governance review. Details of how to apply for the data can be found here: [https://ukmsregister.org/Research/WorkingWithUs] (https:/ukmsregister.org/Research/WorkingWithUs).

## Appendix

Sec Fig. 3.
